# Supplementary material for: Green electrosynthesis of 3,3’-diamino-4,4’-azofurazan energetic materials coupled with energy-efficient hydrogen production over Pt-based catalysts
Source: Nat Commun. 2023 Dec 9;14:8146. doi: 10.1038/s41467-023-43698-x (PMC10709341; doi:10.1038/s41467-023-43698-x)
Supplement: Supplementary file 1 — Supplementary Information files [file 41467_2023_43698_MOESM1_ESM.pdf]

Green electrosynthesis of 3,3'-diamino-4,4'-azofurazan energetic materials coupled with energy-efficient hydrogen production over Pt-based catalysts

Jiachen Li, Yuqiang Ma, Cong Zhang, Chi Zhang, Huijun Ma, Zhaoqi Guo, Ning Liu, Ming Xu, Haixia Ma*, Jieshan Qiu**

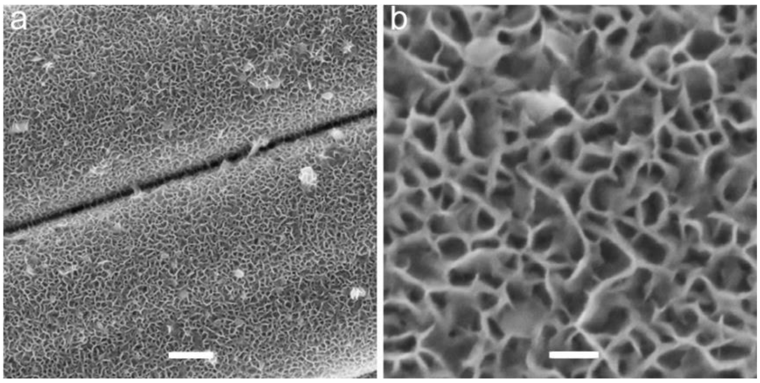

Supplementary Fig. 1. Morphological characterization of CC@WS_2. SEM images of CC@WS_2 at **a** low- and **b** high-resolution (Scale bar: 500 nm for (a) and 100 nm for (b)).

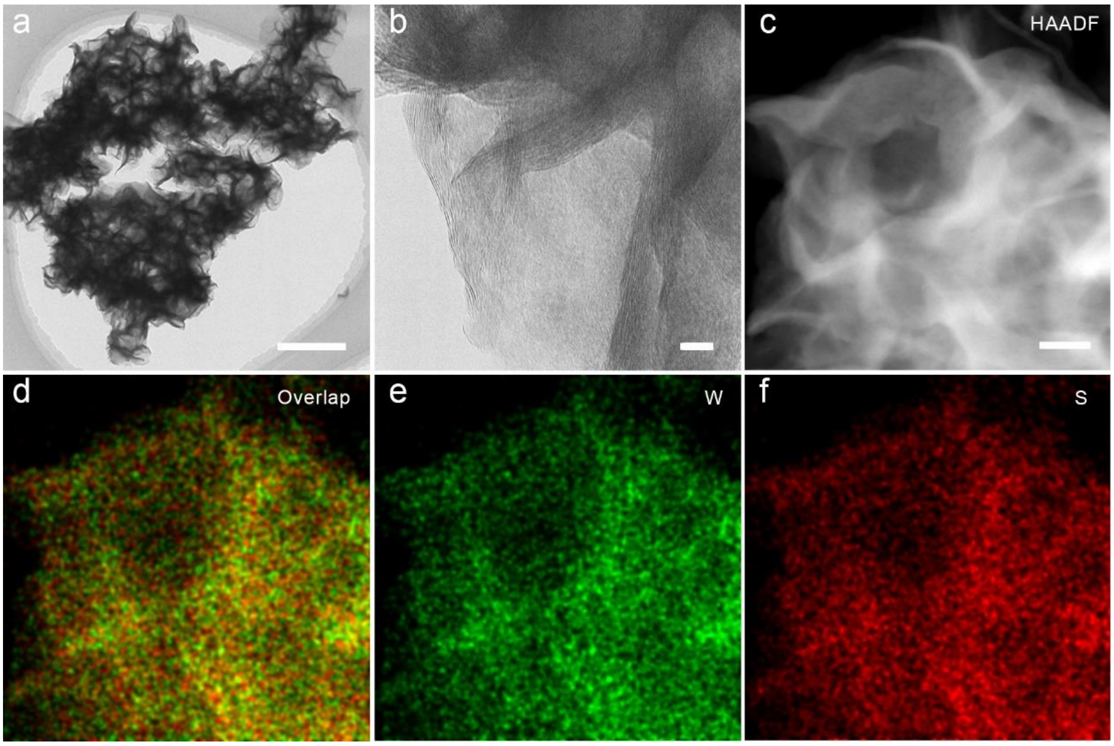

Supplementary Fig. 2. TEM characterizations of WS_2. **a, b** Low and enlarged TEM images (scale bar: 500 nm for (a) and 10 nm for (b)). **c** HAADF-STEM image (scale bar: 50 nm), **d** Overlapped elemental mapping, **e, f** elemental mapping of W and S.

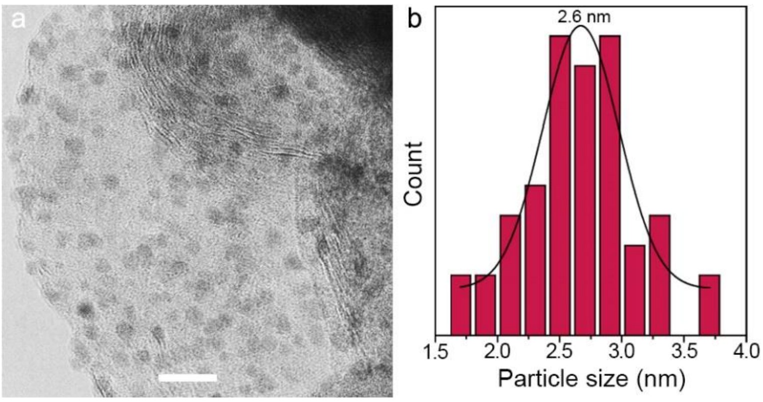

Supplementary Fig. 3. **a** TEM image of $WS_2/Pt_{1,n}$ (Scale bar: 10 nm). **b** Corresponding particle size distribution diagram.

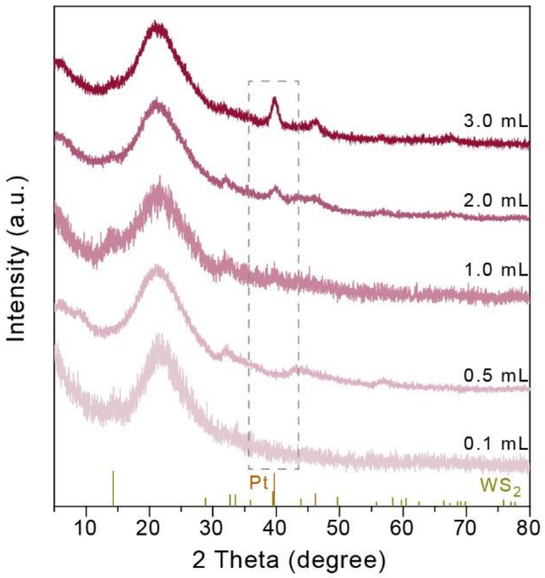

Supplementary Fig. 4. XRD patterns of CC@WS_2/$Pt_{1,n}$ with different Pt contents.

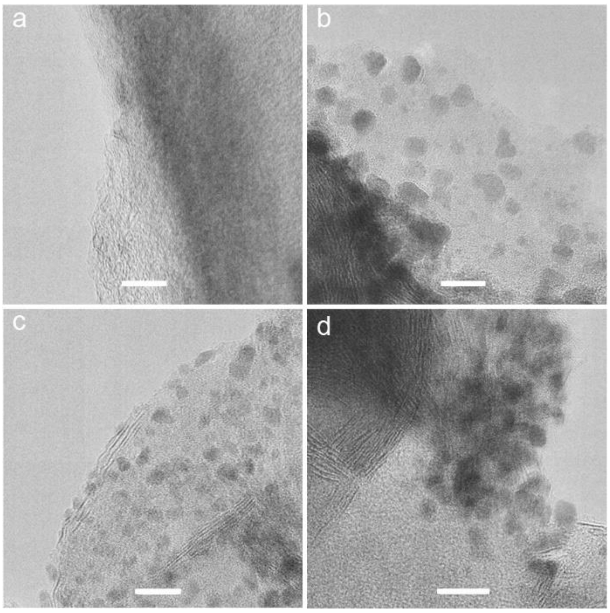

Supplementary Fig. 5. TEM images of CC@WS_2/$Pt_{1,n}$ with different Pt content: **a** 0.1 mL, **b** 0.5 mL, **c** 1.0 mL, and **d** 3.0 mL (scale bar: 20 nm for (a), 10 nm for (b–d)).

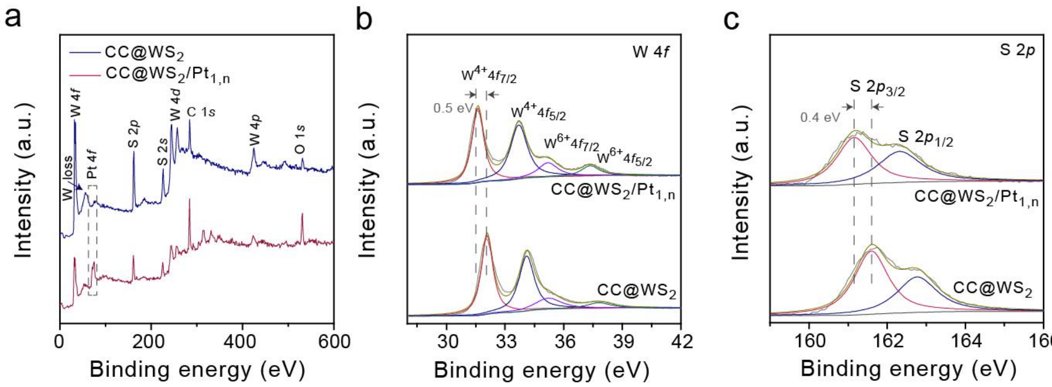

Supplementary Fig. 6. XPS spectra of CC@WS_2 and CC@WS_2/$Pt_{1,n}$. **a** Survey spectra, **b** W 4*f*, **c** S 2*p* signals.

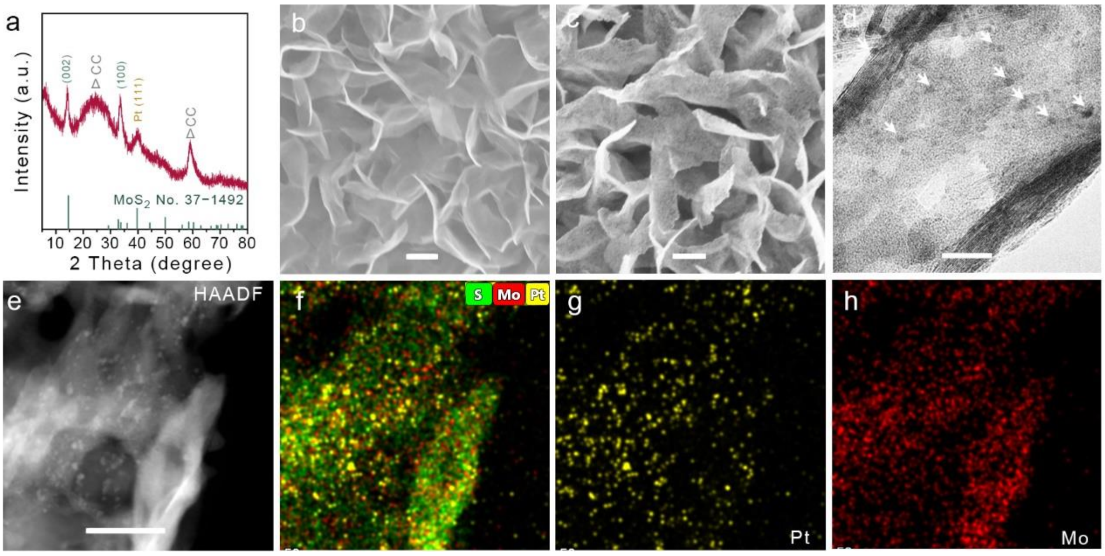

Supplementary Fig. 7. The morphological and structural characterizations of CC@MoS_2 and CC@MoS_2/$Pt_{1,n}$. **a** XRD pattern of CC@MoS_2/$Pt_{1,n}$. **b, c** SEM images of CC@MoS_2 and CC@MoS_2/$Pt_{1,n}$ (scale bar: 200 nm for **b** and **c**). **d** TEM images of CC@MoS_2/$Pt_{1,n}$ (scale bar: 20 nm). **e~h** HAADF-STEM image and elemental mappings of Mo, S, and Pt on CC@MoS_2/$Pt_{1,n}$ (scale bar: 50 nm).

The morphological and structural characterizations of CC@MoS_2 and CC@MoS_2/$Pt_{1,n}$ were performed and shown in Supplementary Fig. 7. XRD pattern revealed the structure of MoS_2 substrate still stable after the deposition of $Pt_{1,n}$ (Fig. 7a). In addition to the diffraction peaks for MoS_2, a distinct peak located at ~40° was attributed to the Pt (111) phase of $Pt_{1,n}$, indicating the successful loading of $Pt_{1,n}$ on CC@MoS_2 NSs. SEM image showed the cross-linking of MoS_2 NSs with smooth surface. After the deposition of $Pt_{1,n}$, the rough surface of MoS_2 NSs was observed without the large Pt nanoparticles, suggesting that the ultrasmall size of $Pt_{1,n}$ were uniformly dispersed on the MoS_2 NSs (Fig. 7b, c). In addition, the TEM and HRTEM images showed that the ultrasmall $Pt_{1,n}$ (direction of arrow) were loaded on the corrugated MoS_2 NSs (Fig. 7d). The corresponding HAADF-STEM image and elemental mappings showed homogeneously dispersed of Mo, S, and Pt on the CC@MoS_2/$Pt_{1,n}$ (Fig. 7e~h)

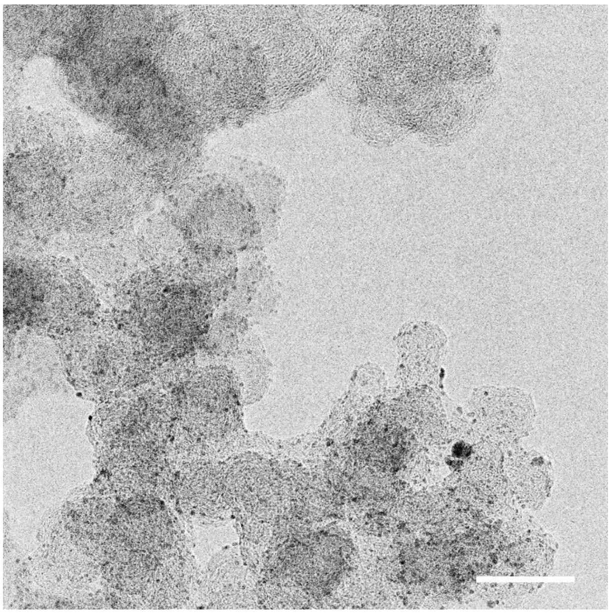

Supplementary Fig. 8. TEM image of 20%Pt/C (scale bar: 50 nm).

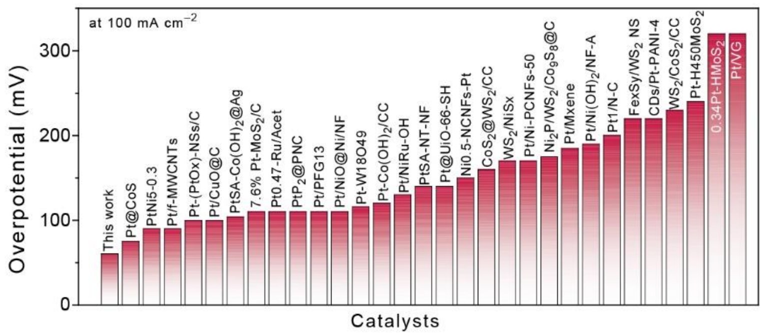

Supplementary Fig. 9. Comparison of the η_{100} of CC@WS_2/$Pt_{1,n}$ with he reported state-of-the-art WS_2 or Pt-based catalysts.

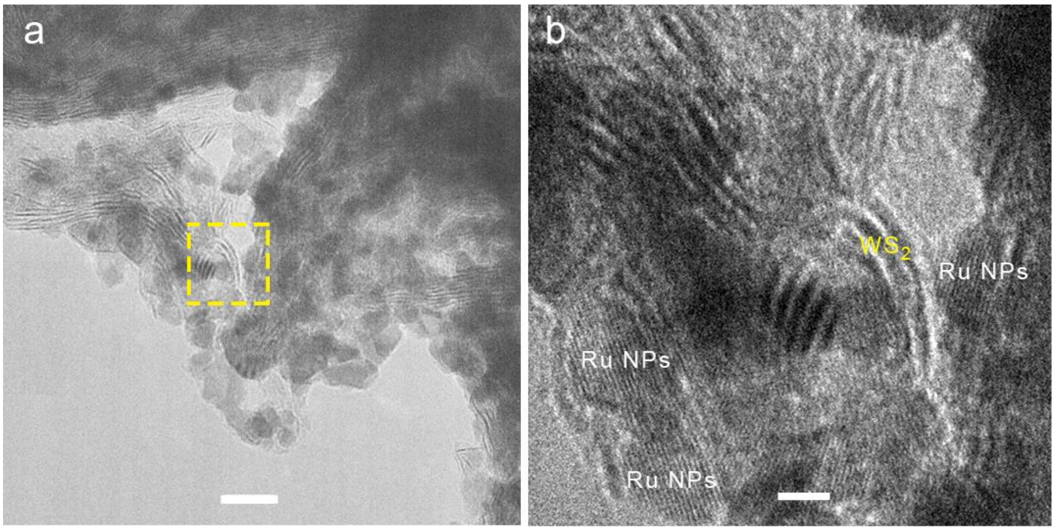

Supplementary Fig. 10. **a** Low and **b** High-resolution TEM images of CC@WS_2/Pt NPs.

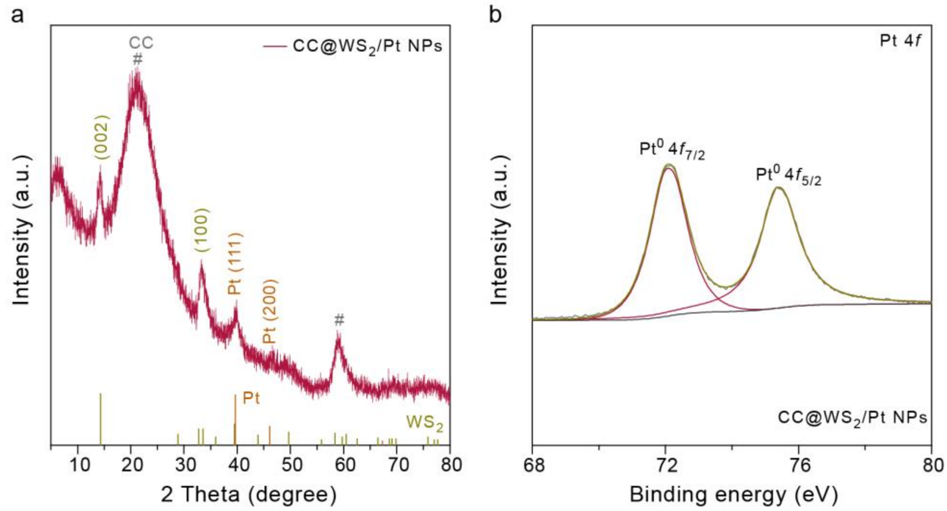

Supplementary Fig. 11. **a** XRD pattern of CC@WS_2/Pt NPs. **b** High-resolution XPS spectrum of Pt 4*f* signal for CC@WS_2/Pt NPs.

The XRD pattern of CC@WS_2/Pt NPs showed that most of the diffraction peaks were consistent with the WS_2 card (JCPDS no. 08–0237), except for two obvious diffraction peaks of 21.3° and 59.1° attributed to the CC substrate. In addition, the distinct Pt (111) and Pt (200) planes detected at 39.8° and 46.6° were ascribed to the Pt NPs (JCPDS no. 04–0802), indicating the successful preparation of Pt NPs on the WS_2 NSs (Supplementary Fig. 11a). The high-resolution XPS of Pt 4*f* signal for CC@WS_2/Pt NPs was performed and shown in Supplementary Fig. 11b. The Pt 4*f* XPS spectrum can be deconvoluted into two peaks, in which the peaks centered at 72.1 and 75.4 eV belong to the Pt–Pt species in Pt NPs, further confirming the successful loading of Pt NPs on CC@WS_2 NSs[1].

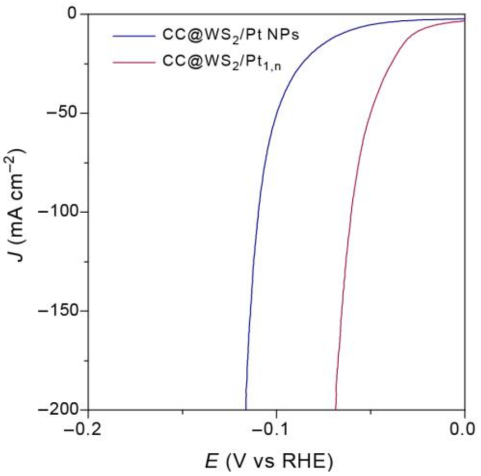

Supplementary Fig. 12. LSV curves of CC@WS_2/$Pt_{1,n}$ and CC@WS_2/Pt NPs.

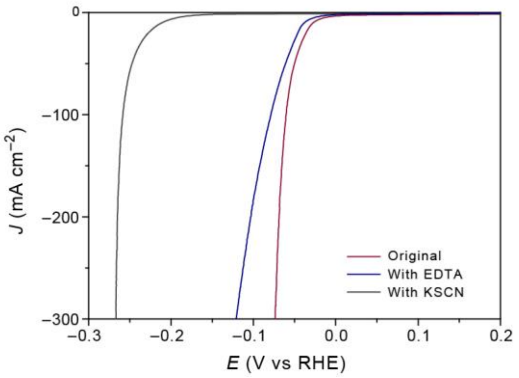

Supplementary Fig. 13. Catalyst poisoning experiment for CC@WS_2/$Pt_{1,n}$ with the addition of 10 mM EDTA or KSCN.

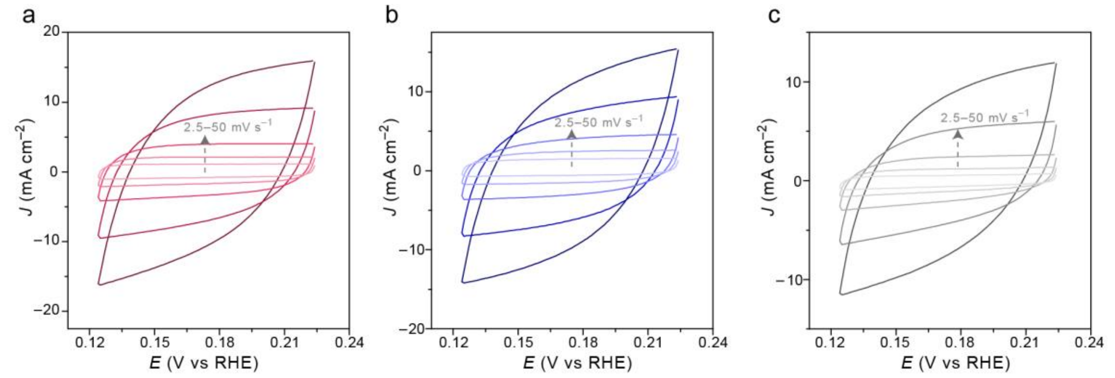

Supplementary Fig. 14. CV curves of **a** CC@WS_2/$Pt_{1,n}$, **b** CC@WS_2, and **c** CC@Pt/C over non-Faradic potential range at scan rates of 2.5, 5, 10, 25, and 50 mV s^{-1}.

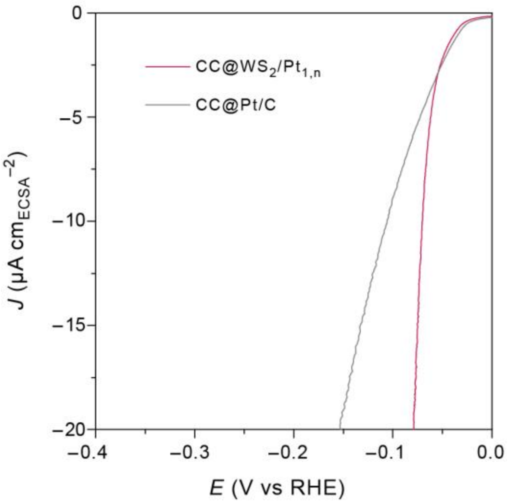

Supplementary Fig. 15. LSV curves of CC@WS_2/$Pt_{1,n}$ and CC@Pt/C, the current density was normalized to the ECSA.

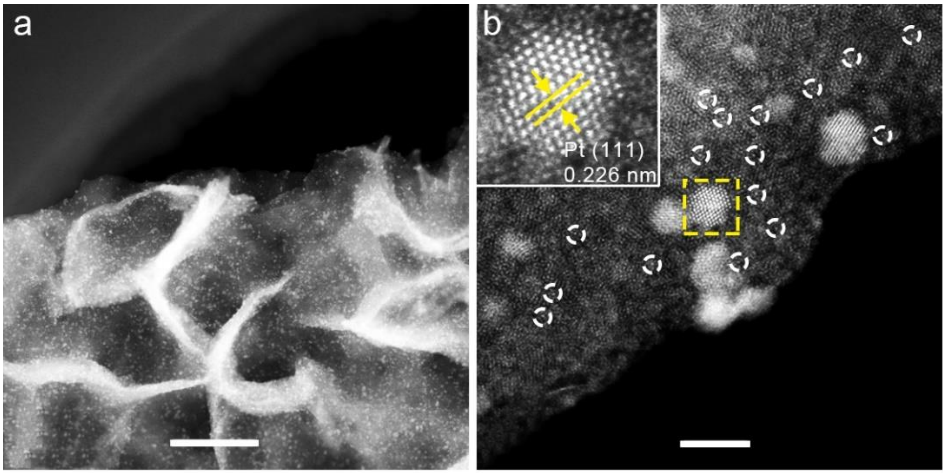

Supplementary Fig. 16. **a** Low and **b** high-resolution HAADF-STEM images of $WS_2/Pt_{1,n}$ after CP test (Scale bar: 100 nm for **a** and 5 nm for **b**).

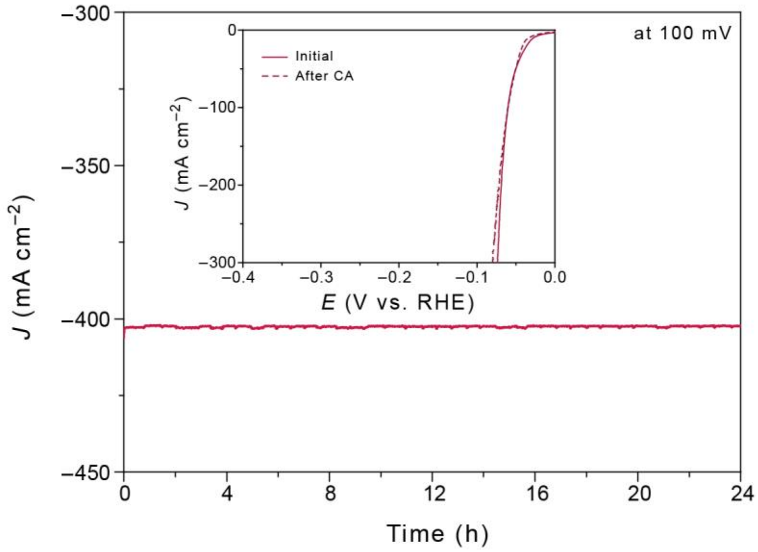

Supplementary Fig. 17. Long-term stability CA test of CC@WS_2/$Pt_{1,n}$. (inset: LSV curves before and after the CA test).

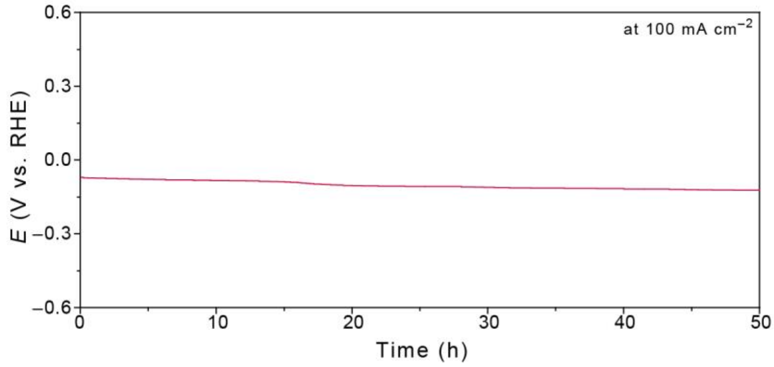

Supplementary Fig. 18. Long-term chronopotentiometry test of CC@WS_2/$Pt_{1,n}$ at a current density of 100 mA cm^{-2}.

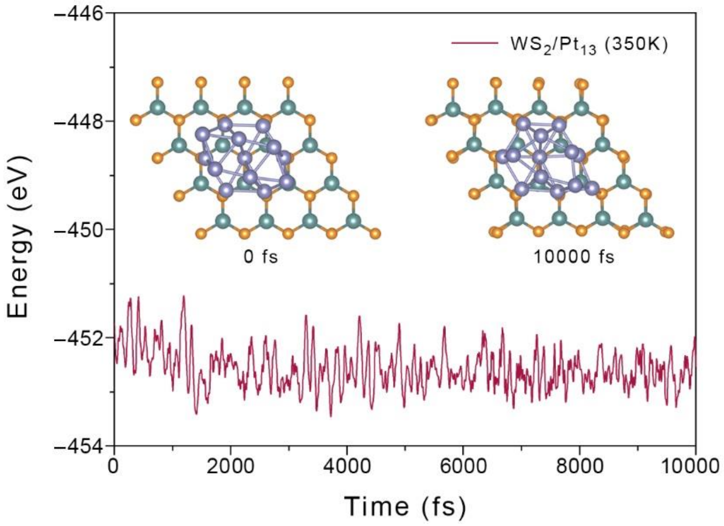

Supplementary Fig. 19. AIMD simulation trajectories as well as the obtained optimal structures for WS_2/Pt_{13}.

We included the results of *ab initio* molecular dynamics (AIMD) simulations by using VASP code as a complement to evaluate the dynamical stability of the Pt_{13} clusters on WS_2. The AIMD calculations were run under an NVT ensemble at T = 350 K for t = 1 ps total simulation time for WS_2/Pt_{13}. From the optimized structure, no obvious changes in geometries or energies were observed, evidencing the good stability of the WS_2/Pt_{13} composite.

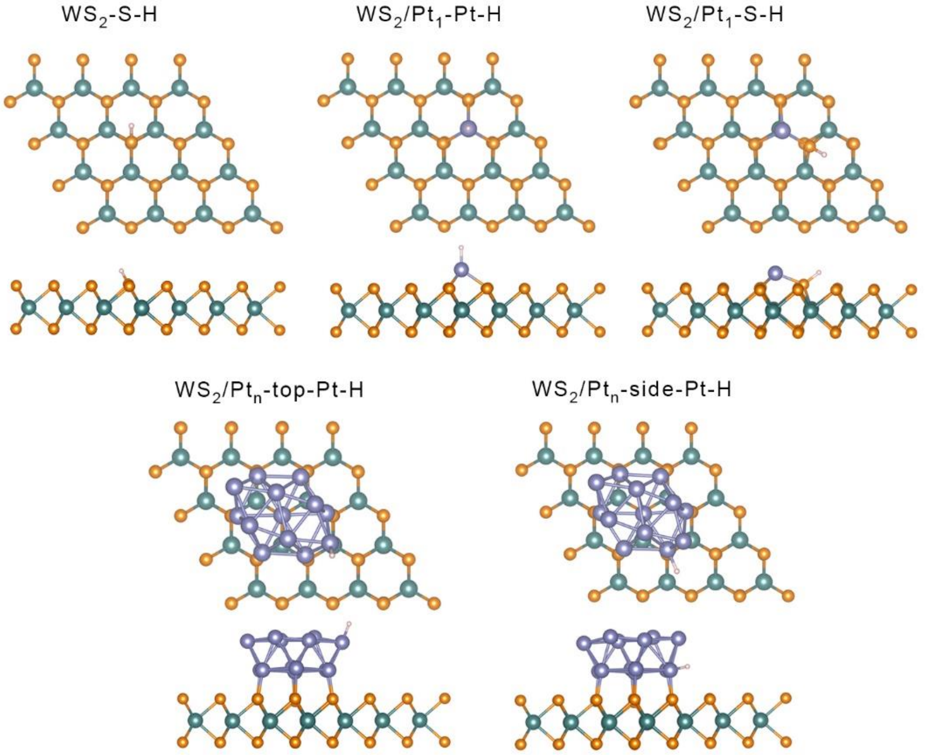

Supplementary Fig. 20. Structural models of H atom adsorbed on S and Pt sites of WS_2, WS_2/Pt_1 and WS_2/Pt_n, respectively.

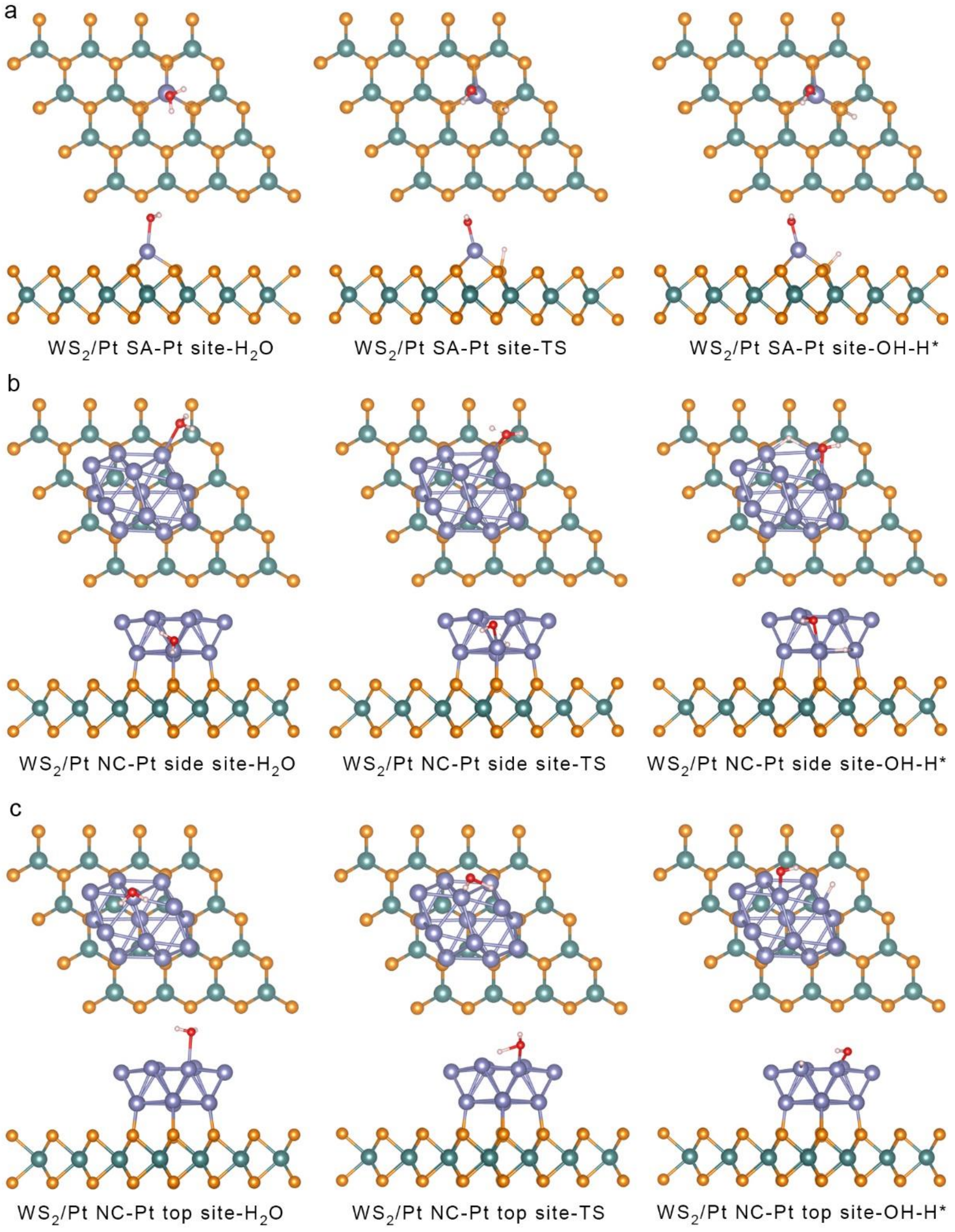

Supplementary Fig. 21. Structural models of H_2O, OH*, and H* adsorbed on different sites of WS_2/Pt_1 and WS_2/Pt_n, respectively.

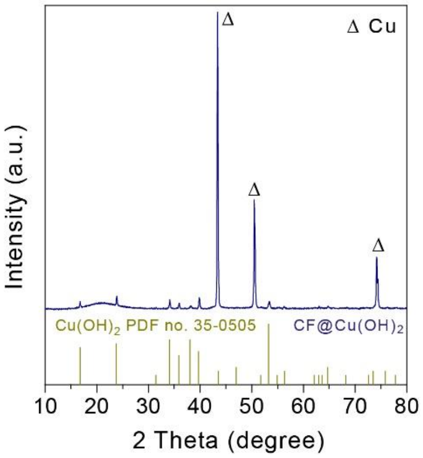

Supplementary Fig. 22. XRD pattern of CF@$Cu(OH)_2$ NWs.

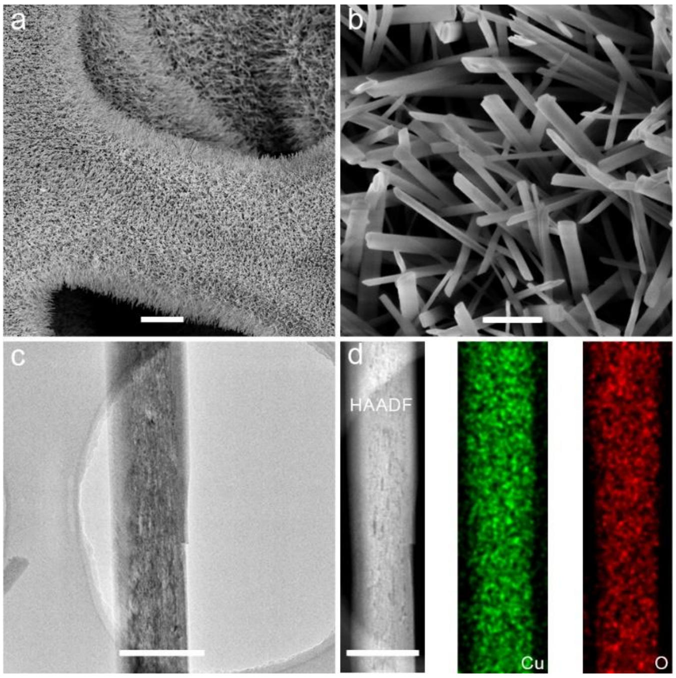

Supplementary Fig. 23. Morphological characterizations of CF@Cu(OH)$_2$ NWs. SEM images CF@Cu(OH)$_2$ NWs at **a** low and **b** high-resolution (Scale bar: 20 μm for (a) and 1 μm for (b)). **c** TEM and **d** HAADF-STEM elemental mappings of Cu(OH)$_2$ NWs (Scale bar: 500 nm for **c** and **d**).

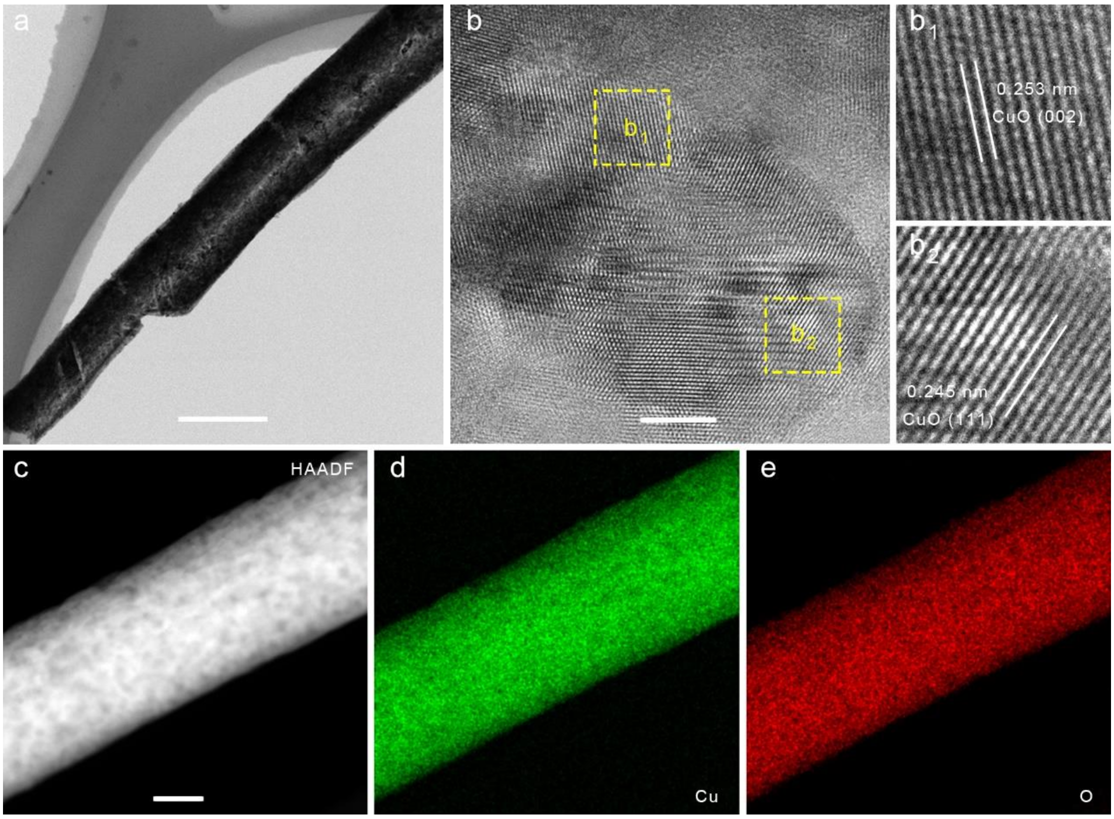

Supplementary Fig. 24. Morphological characterizations of CuO NWs. **a, b** Low and high-resolution TEM images of CuO NWs **b1** and **b2**: two enlarged regions of **b**). **c** HAADF-STEM image and **d, e** elemental mappings of Cu and O signals for CuO (scale bar: 0.5 μm for **a**, 5 nm for **b**, and 50 nm for **c**).

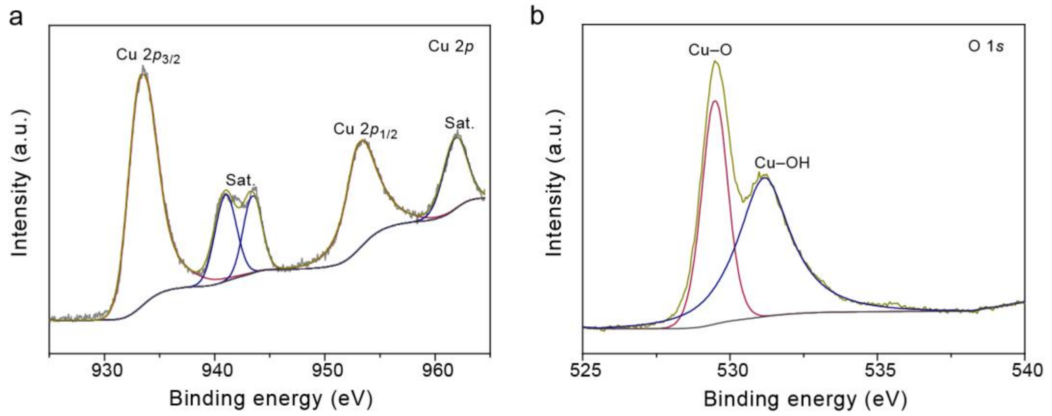

Supplementary Fig. 25. High-resolution XPS spectrum of **a** Cu 2p and **b** O 1s signals for CF@CuO NWs.

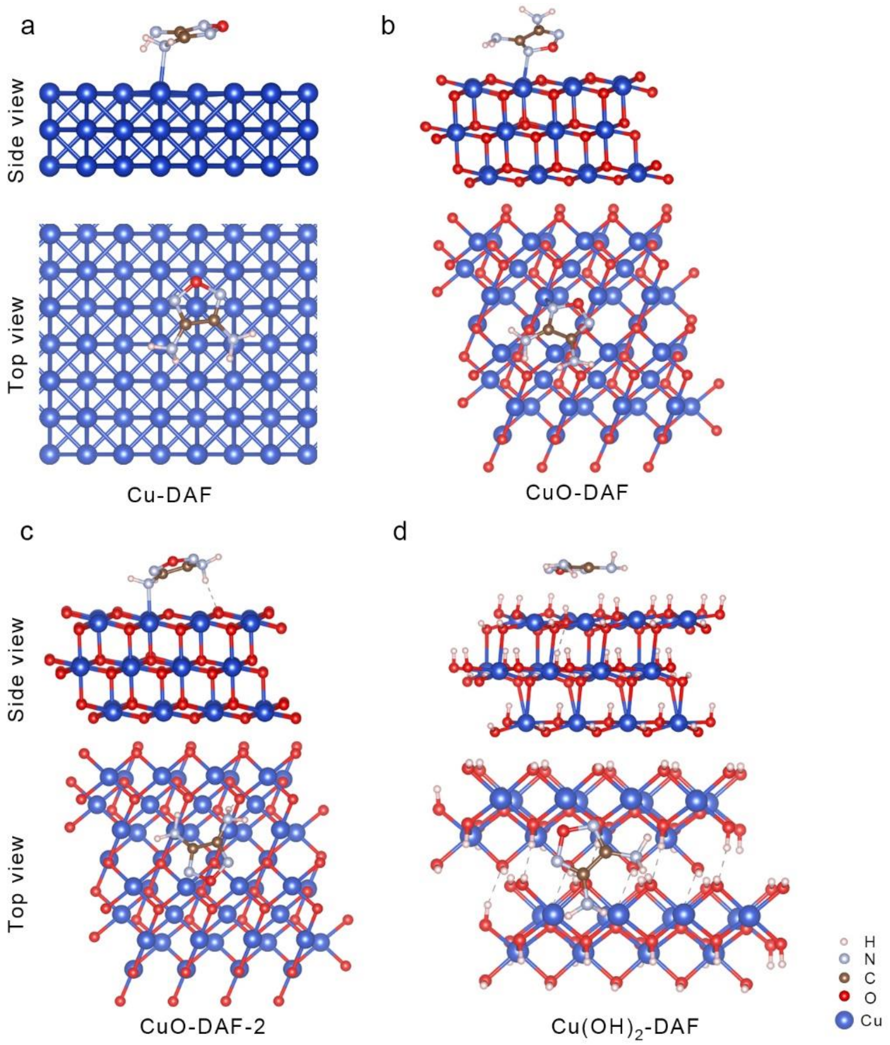

Supplementary Fig. 26. Structural models of **a** Cu (100)-DAF, **b** CuO (111)-DAF, **c** CuO (111)-DAF-2, and **d** $Cu(OH)_2$ (002)-DAF.

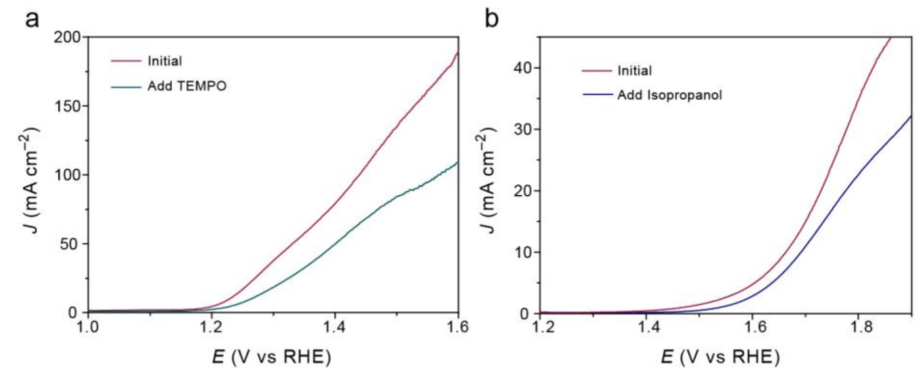

Supplementary Fig. 27. Free radical quenching tests. LSV curves of DAF oxidation reaction in 0.20 M DAF+1.0 M KOH with or without the addition of (a) TEMPO (0.6 mmol) free radical scavengers on CF@CuO NWs and (b) isopropanol (5.0 mL) free radical scavengers on Pt film.

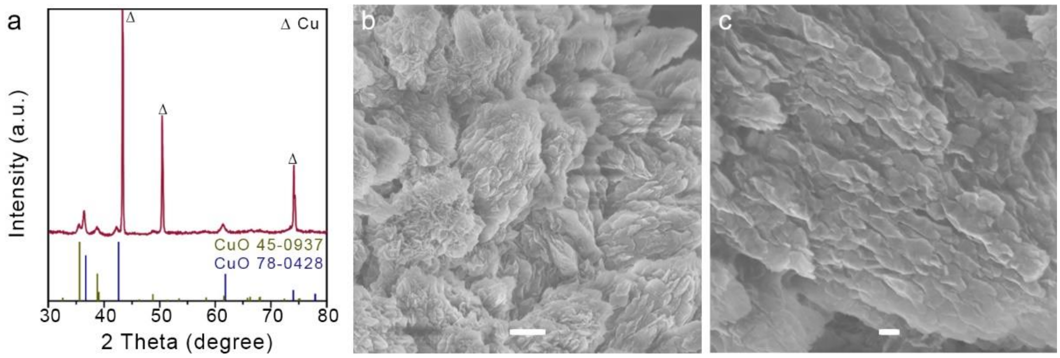

Supplementary Fig. 28. Morphological and structural characterizations of CF@CuO NWs after CP test. **a** XRD pattern, **b, c** SEM images of CC@CuO NWs after CP test with the addition of DAF (scale bar: 1 μm for **b** and 200 nm for **c**).

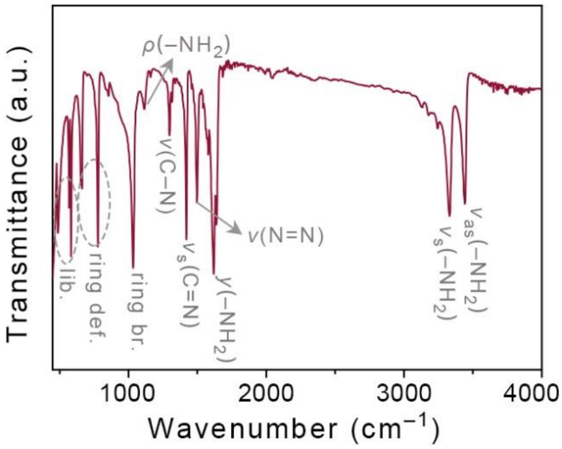

Supplementary Fig. 29. FTIR spectrum of DAAzF EMs.

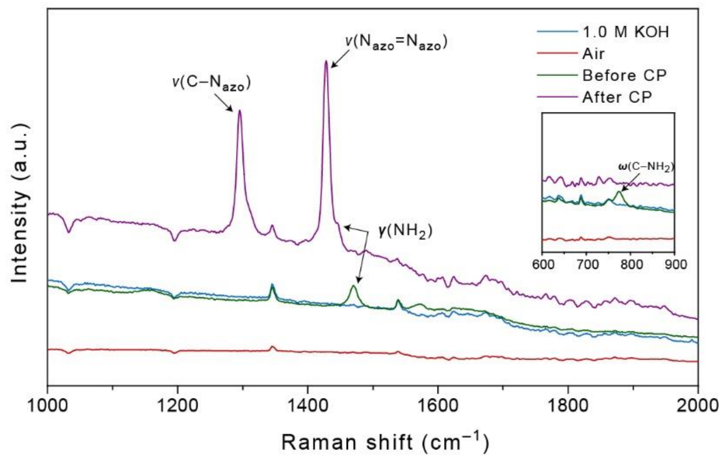

Supplementary Fig. 30. Raman spectra of anode electrolyte before and after long-term CP test of coupling system, the Raman spectra of pure 1.0 M KOH and air were set for comparison (inset: Raman spectra at lower frequency).

the Raman spectra was performed to eliminate the influence of water molecules toward other characteristic peaks in FTIR and further confirmed the successful transform of DAF to DAAzF EMs via N–N oxidative-coupling pathway. The formation of the azo compounds is easily confirmed by comparing the Raman spectra of the reaction products with that of the starting material. Compared with initial DAF alkaline solution, two dominate peaks located at 1295 and 1428 cm^{-1} were detected after CP test, corresponding to the $C–N_{azo}$ stretching and the stretching mode of the azo group, respectively[2–4]. In addition, the decreased intensity of $\gamma(NH_3)$ and $\omega(C-NH_2)$ stretching modes indicated the dehydrogenation of amino group and then involved N–N oxidative-coupling process to form DAAzF EMs[5]. Together with the ^{13}C NMR and FTIR analysis, the anodic product could be identified to the pure DAAzF EMs through the N–N oxidative-coupling of DAF starting material.

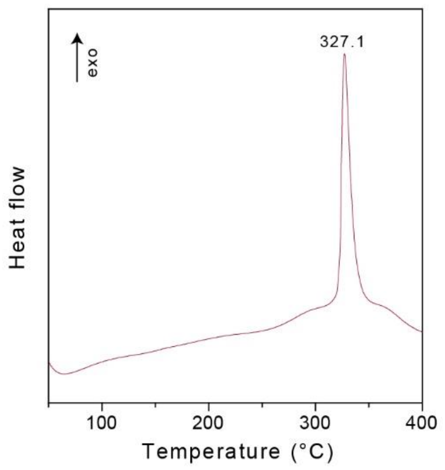

Supplementary Fig. 31. DSC curve of DAAzF EMs.

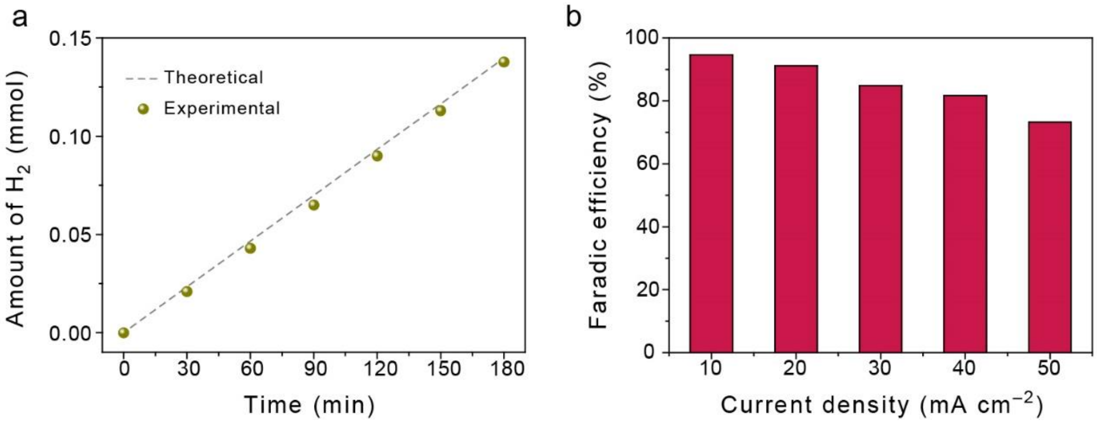

Supplementary Fig. 32. **a** FE of HER at the constant current density of 10 mA cm^{-2} and **b** FE of DAFOR at different current densities.

Supplementary Table 1. EXAFS fitting parameters at the Pt L_3-edge for MoS_2-Pt_1 and Pt foil reference

**Sample**	Shell	N [a]	R (Å) [b]	σ^2 (Å^2·10^{-3}) [c]	ΔE^0 (eV) [d]	R factor (%)
MoS_2-Pt_1	Pt−S	3.1(±0.6)	2.31(±0.04)	0.7	4.6	0.9
	Pt-Mo	1.1(±0.3)	2.80(±0.20)	6.7	-2.0	
Pt foil (25 °C)	Pt−Pt (in metallic state Ru)	12	2.76	4.1	8.1	0.06

[a] N: coordination number; [b] R: bond distance; [c] σ^2: Debye-Waller factor; [d] ΔE_0: the inner potential correction.

R factor: goodness of fit. S_0^2, 0.779, was obtained from the experimental EXAFS fitting over Pt foil reference with known crystallographic value, which was then used to all the samples.

Supplementary Table 2. A comparison of the HER activity of CC@WS_2/$Pt_{1,n}$ with those of recently reported similar Pt-based electrocatalysts at 10 mA cm^{-2}.

**Catalysts**	**Electrolytes**	η_{10} **(mV)**	***Ref.***
CC@WS_2/$Pt_{1,n}$	1.0 M KOH	27.1	*This work*
Pt/Ni ASs/C	1.0 M KOH	28.0	*Nano Lett.* **2021**, *21, 22,* 9381–9387
Pt/CoO_x	0.5 M H_2SO_4	28.0	*Appl. Catal. B. Environ.,* **2022**, *314,* 121503
Pt@CoS	1.0 M KOH	28.0	*Appl. Catal. B. Environ.,* **2022**, *315,* 121534
Pt-SAs/$MoSe_2$	1.0 M KOH	29.0	*Nat. Commun.* **2021**, *12,* 3021
Pt_{SA}-Co(OH)$_2$@Ag	1.0 M KOH	29.0	*Energy Environ. Sci.,* **2020**, *13,* 3082–3092
NF-Na-Fe-Pt	1.0 M KOH	31.0	*Appl. Catal. B. Environ.,* **2021**, *297,* 120395
Pt/$Ti_3C_2T_x$	0.1 M $HClO_4$	32.7	*Nano Lett.* **2019**, *19, 8,* 5102–5108
Pt/Mxene	0.5 M H_2SO_4	34.0	*Adv. Funct. Mater.* **2022**, 2110910
Pt@Cu	1.0 M KOH	35.0	*Adv. Funct. Mater.* **2021**, *31,* 2105579
Pt@DG	1.0 M KOH	37.0	*J. Am. Chem. Soc.* **2022**, *144, 5,* 2171–2178
PtNi-NC-900	1.0 M KOH	37.4	*J. Mater. Chem. A,* **2022**, *10,* 13727–13734
Pt_1/OLC	0.5 M H_2SO_4	38.0	*Nat. Energy* **2019**, *4,* 512–518
N,Pt/MoS_2	1.0 M KOH	38.0	*Energy Environ. Sci.,* **2022**,*15, 1201–1210*
PtSi	1.0 M KOH	38.0	*Adv. Energy Mater.* **2022**, 2200293
CoPt-Pt_{SA}/NDPCF	1.0 M KOH	38.0	*Adv. Funct. Mater.* **2022**, 2205920
$Ti_3C_2T_x$-Pt_{SA}	0.5 M H_2SO_4	38.0	*Nano Lett. 2022, 22, 1398–1405*
$PtSe_2$/Pt	1.0 M KOH	42.0	*Angew. Chem. Int. Ed.* **2021**, *60,* 23388–23393
F-SnO_2@Pt	0.5 M H_2SO_4	42.0	*ACS Nano* **2022**, *16,* 1625–1638
Pt@WO_3@RGO	0.5 M H_2SO_4	42.0	*Small* **2021**, 2102159
PtCo-NC-900	0.5 M H_2SO_4	45.5	*J. Phys. Chem. Lett.* **2022**, *13,* 5195–5203
Pt_1/N-C	1.0 M KOH	46.0	*Nat. Commun.* **2020**, *11,* 1029
Pt-Ru SWNT	0.1 M KOH	46.0	*Appl. Catal. B. Environ.,* **2022**, *315,* 121541
PtRu@C_2N	0.5 M H_2SO_4	52.0	*Chem. Eng. J.* **2022**, *428,* 131085
Pt@UiO-66-SH	0.5 M H_2SO_4	57.0	*Inorg. Chem.* **2022**, *61, 34,* 13271–13275
Pt@Nb_2CT_x	1.0 M KOH	65.1	*Adv. Sci.* **2021**, 2102207
Pt@MoS_2	0.5 M H_2SO_4	67.4	*Nano Energy* **2022**, *94,* 106913
Pt@VS_2	0.5 M H_2SO_4	77.0	*ACS Nano* **2020**, *14,* 5600–5608
Pt@MoS_2	1.0 M KOH	88.43	*Nano Energy* **2021**, *84,* 105898
0.34Pt-HMoS_2	1.0 M KOH	112	*Small* **2021**, 2104824
Pt/VG	1.0 M KOH	124	*Adv. Funct. Mater.* **2022**, 2203067

Supplementary Table 3. A comparison of the HER activity of CC@WS_2/$Pt_{1,n}$ with those of recently reported Pt- and WS_2-based electrocatalysts at 100 mA cm^{-2}.

Catalysts	Electrolytes	η_{100} (mV)	*Ref.*
CC@WS_2/$Pt_{1,n}$	1.0 M KOH	60.4	*This work*
Pt@CoS	1.0 M KOH	75	*Appl. Catal. B Environ*. **2022**, *315*, 121534
PtNi5-0.3	1.0 M KOH	90	*Adv. Funct. Mater*. **2021**, *31*, 2008298
Pt/f-MWCNTs	0.5 M H_2SO_4	90	*Nano Energy*, **2019,** *63*, 103849
Pt-(PtOx)-NSs/C	0.5 M H_2SO_4	100	*ChemSuschem*, 2018, *11*, 2388–2401
Pt/CuO@C	1.0 M KOH	100	*Chem. Eng. J*, **2023**, *455*, 140856
PtSA-Co$(OH)_2$@Ag	1.0 M KOH	104	*Energy Environ. Sci*., **2020**, *13*, 3082–3092
7.6% Pt-MoS_2/ C	0.5 M H_2SO_4	110	*ACS Sustain. Chem. Eng*. **2018**, *6*, 7704–7714
$Pt_{0.47}$-Ru/Acet	1.0 M KOH	110	*Chem. Eng. J*, **2022**, *448*, 137611
PtP_2@PNC	0.5 M H_2SO_4	110	*iScience*, **2020**, *23*, 101793
Pt/PFG13	0.5 M H_2SO_4	110	*Int. J Hydr. Energy*, **2019**, *44*, 24151–24161
Pt/NiO@Ni/NF	1.0 M KOH	110	*ACS Catal*. **2018**, *8*, 8866–8872
Pt-$W_{18}O_{49}$	0.5 M H_2SO_4	116	*Sci. China Mater*. **2022**, *65*, 3435–3441
Pt-Co$(OH)_2$/CC	1.0 M KOH	120	*ACS Catal*. **2017**, *7*, 7131–7135
Pt/NiRu-OH	1.0 M KOH	130	*Appl. Catal. B Environ*. **2020**, *269*, 118824
Pt@UiO-66-SH	1.0 M KOH	140	*Inorg. Chem*. **2022**, *61*, 34, 13271–13275
PtSA-NT-NF	1.0 M PBS	140	*Angew. Chem. Int. Ed*. **2017**, *56*, 13694–13698
Ni0.5-NCNFs-Pt	0.5 M H_2SO_4	150	*J Colloid and Interface Sci*., **2018**, *514*, 199–207
CoS_2@WS_2/CC	0.5 M H_2SO_4	160	*J Mater. Chem. A*, **2017**, *5*, 15552–15558
WS_2/NiS_x	1.0 M KOH	170	*In. J Hydr. Energy*, **2022**, *47*, 33643–33651
Pt/Ni-PCNFs-50	1.0 M KOH	170	*Chin. Chem. Letter*, **2023**, *34*, 107359
Ni_2P/WS_2/Co_9S_8@C	1.0 M KOH	175	*Chem. Eng. J*, **2022**, *446*, 136961
Pt/Mxene	0.5 M H_2SO_4	185	*Adv. Funct. Mater*. **2022**, 2110910
Pt/Ni$(OH)_2$/NF-A	1.0 M KOH	190	*J. Mater. Chem. A*, **2021**, *9*, 16427–16435
Pt_1/N-C	1.0 M KOH	~200	*Nat. Commun*. **2020**, *11*, 1029
FexSy/WS_2 NS	0.5 M H_2SO_4	220	*Energy Fuels*, **2022**, *36*, 4888–4894
CDs/Pt-PANI-4	1.0 M KOH	220	*Appl. Catal. B Environ*. **2019**, *257*, 117905
WS_2/CoS_2/CC	1.0 M KOH	230	*ACS Sustain. Chem. Eng*. **2020**, *8*, 4474–4480
Pt-H450MoS_2	0.5 M H_2SO_4	240	*Small*, **2022**, *18*, 2104824
4.78-Pt/V_8G@C	1.0 M KOH	240	*Appl. Catal. A General*, **2022**, *633*, 118512
Ni-Mo@Pt-0.03	1.0 M KOH	242	*New J Chem*. **2021**, *45*, 16313–16318
V0.065-WS_2/CC	1.0 M KOH	260	*Chem. An Asian J*, **2018**, *13*, 1438
WS_2/$CoSe_2$	0.5 M H_2SO_4	300	*J. Industrial Eng. Chem*. **2018**, *65*, 167–174
0.34Pt-HMoS_2	1.0 M KOH	~320	*Small* **2021**, 2104824
Pt/VG	1.0 M KOH	~320	*Adv. Funct. Mater*. **2022,** 2203067
CeO_2/WS_2/CC	1.0 M KOH	400	*J Power Sources*, **2022**, *521*, 230948
WS_2	0.5 M H_2SO_4	490	*Flatchem*. **2021,** *29*, 100278

Supplementary Table 4. Comparison of the ΔG_{H*} on various sites of reported pristine WS_2 catalysts.

Catalysts	H*-adsorption sites	ΔG_{H*} (eV)	References
$WS_2/Pt_{1,n}$	2H-WS_2-S site<br>2H-WS_2/Pt SA-S site	2.069<br>1.648	*This work*
Co:WS_2	2H-WS_2-S sites<br>1T-WS_2-S sites	~2.4<br>~0.9	*Energy Environ. Sci., 2018, 11, 2270–2277*
P, Ni-WS_2	2H-WS_2-W sites	2.447	*Nano Energy 2019, 55 193–202*
WS_2/Co_9S_8	2H-WS_2	2.24	*Adv. Funct. Mater. 2022, 2112362*
WS_2/CC	2H-WS_2-S sites	2.4	*ChemCatChem 2019, 11, 2667 –2675*
W_2C@WS_2	2H-WS_2	2.26	*Adv. Funct. Mater. 2017, 27, 1605802*
Te-WS_2	2H-WS_2-S sites<br>2H-WS_2-W sites<br>2H-WS_2-hollow sites	2.27<br>2.42<br>2.28	*J. Catal. 2020, 382, 204–211*
Nb-WS_2	2H-WS_2	2.185	*ACS Appl. Mater. Interfaces 2019, 11, 38, 34862–34868*
Fe-WS_2	2H-WS_2	2.304	*ACS Appl. Mater. Interfaces 2021, 13, 16, 19406–19413*
WS_2@Graphene	2H-WS_2	~2.4	*Adv. Mater. 2020, 32, 2002584*
$Ni_2P/WS_2/Co_9S_8$@C	2H-WS_2	2.12	*Chem. Eng. J. 2022, 446, 136961*
P-WS_2	2H-WS_2-S sites	2.17	*Nano Res. 2022, 15, 2855–2861*
Co_9S_8/2H-WS_2@NF	2H-WS_2	2.09	*ChemCatChem 2022, 14, e202101553*
Co, N-WS_2	2H-WS_2	2.33	*Nano Res. 2022, 15, 1993–2002*

Supplementary Table 5. A comparison of cell voltages of the electrosynthesis of DAAzF coupled with OWS for CC@WS_2/$Pt_{1,n}$||CF@CuO NWs at 10 mA cm^{-2} with those of recently reported state-of-the-art electrocatalysts.

**Catalysts**	**Electrolytes**	***E_{10}* (V)**	***Ref.***
CC@WS_2/$Pt_{1,n}$||CF@CuO NWs	1.0 M KOH//1.0 M KOH+0.2 M DAF	1.26	*This work*
Pt/TiO_2/*β*-$Ni(OH)_2$||Pt/TiO_2/*β*-$Ni(OH)_2$	1.0 M KOH//1.0 M KOH	1.37	*Appl. Catal. B: Environ.* **2022**, *316*, 121654
Ru-$NiCo_2O_4$||Ru-$NiCo_2O_4$	1.0 M KOH//1.0 M KOH	1.45	*Appl. Catal. B: Environ.* **2022**, *305*, 121081
Ir-NiCoLDH||Ir-NiCoLDH	1.0 M KOH//1.0 M KOH	1.45	*J. Mater. Chem. A*, **2020**, *8*, 9871–9881
v_s-Ru-Ni_9S_8||v_s-Ru-Ni_9S_8	1.0 M KOH//1.0 M KOH	1.47	*Appl. Catal. B: Environ.* **2022**, *310*, 121356
RuV–CoNiP/NF||RuV–CoNiP/NF	1.0 M KOH//1.0 M KOH	1.47	*J. Mater. Chem. A*, **2021**, *9*, 26852–26860
$Ru_{0.5}Ir_{0.5}$||$Ru_{0.5}Ir_{0.5}$	1.0 M KOH//1.0 M KOH	1.48	*Chem. Eng. J.* **2022**, *450*, 137909
Pt–IrO_2/CC||IrO_2/CC	1.0 M KOH//1.0 M KOH	1.49	*Adv. Energy Mater.* **2020**, 2001600
IrO_2/V_2O_5||IrO_2/V_2O_5	1.0 M KOH//1.0 M KOH	1.49	*Adv. Sci.* **2022**, *9*, 2104636
Ir-C||Ir-C	1.0 M KOH//1.0 M KOH	1.495	*ACS Catal. 2021*, *11*, 1179–1188
$Pd_{44}Pt_{30}Ir_{26}$ ASNSs/C||$Pd_{44}Pt_{30}Ir_{26}$ ASNSs/C	1.0 M KOH//1.0 M KOH	1.50	*ACS Catal.* **2022**, *12*, 5305–5315
NCO@RuO_2-NCs||NCO@RuO_2-NCs	1.0 M KOH//1.0 M KOH	1.50	*Chem. Eng. J.* **2022**, *446*, 137037
IrW/WO_3-NS-A||IrW/WO_3-NS-A	0.5 M H_2SO_4//0.5 M H_2SO_4	1.50	*ACS Appl. Energy Mater.* **2022**, *5*, 970–980
Pt–CoFe LDHs||Pt–CoFe LDHs	1.0 M KOH//1.0 M KOH	1.51	*J. Power Sources* **2022**, *532*, 231353
Ir@S–C/rGO||Ir@S–C/rGO	1.0 M KOH//1.0 M KOH	1.51	*J. Mater. Chem. A*, **2021**, *9*, 4176–4183
RuO_2-C-300//RuO_2-C-300	1.0 M KOH//1.0 M KOH	1.52	*Small* **2022**, *18*, 2203778
PtIr/IrO_x-30 NWs/C||PtIr/IrO_x-30 NWs/C	1.0 M KOH//1.0 M KOH	1.52	*Small* **2022**, 2201333
Li-$IrSe_2$|| Li-$IrSe_2$	1.0 M KOH//1.0 M KOH	1.52	*Angew. Chem. Int. Ed.* **2019**, *58*, 14764–14769
La-doped RuO_2||La-doped RuO_2	0.5 M H_2SO_4//0.5 M H_2SO_4	1.53	*Chem. Eng. J.* **2022**, *439*, 135699

Pt–RuO_2@KB||Pt–RuO_2@KB	0.1 M $HClO_4$||0.1 M $HClO_4$	1.54	*J. Mater. Chem. A*, **2022**, *10*, 13241–13246
V-Ni_3FeN/Ni@N-GTs|| V-Ni_3FeN/Ni@N-GTs	1.0 M KOH//1.0 M KOH	1.55	*J. Mater. Chem. A*, **2022**, *10*, 18877
Pt-WO_{3-x}@rGO||Pt-WO_{3-x}@rGO	1.0 M KOH//1.0 M KOH	1.55	*Chem. Eng. J.* **2022**, *431*, 133287
Pt-Cu@Cu_xO NWs/3DF||Pt-Cu@Cu_xO NWs/3DF	0.1 M KOH//0.1 M KOH	1.56	*Nano Energy* **2019**, *59*, 216–228
Ir-WO_3||Ir-WO_3	0.5 M H_2SO_4//0.5 M H_2SO_4	1.56	*Small* **2021**, *17*, 2102078
Ir/Ni_3Fe/rGO||Ir/Ni_3Fe/rGO	1.0 M KOH//1.0 M KOH	1.57	*Chem. Eng. J.* **2023**, *451*, 138548
Ir-BP||Ir-BP	1.0 M KOH//1.0 M KOH	1.57	*Adv. Mater.* **2021**, 2104638
D-RuO_2/TiO_2/TM||D-RuO_2/TiO_2/TM	1.0 M KOH//1.0 M KOH	1.59	*Chem. Eng. J.* **2022**, *431*, 134072
Pt-NiO/Gr-SUS||Pt-NiO/Gr-SUS	1.0 M KOH//1.0 M KOH	1.60	*ACS Nano* **2022**, *16*, 930–938
Ir_1@Co/NC||Ir_1@Co/NC	1.0 M KOH//1.0 M KOH	1.60	*Angew. Chem. Int. Ed.* **2019**, *58*, 11868–11873
$Ir_{0.75}W_{0.25}$||$Ir_{0.75}W_{0.25}$	0.1 M KOH//0.1 M KOH	1.60	*Nanoscale*, **2019**, *11*, 8898–8905
RuO_2–WC NPs||RuO_2–WC NPs	0.5 M H_2SO_4//0.5 M H_2SO_4	1.66	*Angew. Chem. Int. Ed.* **2022**, *61*, e202202519

References

1. Jin, H., Ha, M., Kim, M. G., Lee, J. H. & Kim, K. S. Engineering Pt coordination environment with atomically dispersed transition metal sites toward superior hydrogen evolution. *Adv. Energy Mater.* **13**, 2204213 (2023).
2. Gao, C. *et al.* Initial decomposition step and bimolecular hydrogen transfer of 3, 3′-diamino-4, 4′-azoxyfurazan under high pressure and high temperature. *Combust. Flame* **240**, 111981 (2022).
3. Klapötke, T. M. & Sabaté, C. M. Nitrogen-rich tetrazolium azotetrazolate salts: A new family of insensitive energetic materials. *Chem. Mater.* **20**, 1750–1763 (2008).
4. Hammer, A. *et al.* Azidoformamidinium and guanidinium 5,5′-azotetrazolate salts. *Chem. Mater.* **17**, 3784–3793 (2005).
5. Gao, C. *et al.* Pressure-dependent luminescence and absorption in 3,3′-Diamino-4,4′-azoxyfurazan: Secondary bonding interaction in molecular crystals. *J. Phys. Chem. C* **123**, 8731–8739 (2019).
